# A comparative study between Dydrogesterone alone and combined with Non-Steroidal Anti-Inflammatory Drugs in the treatment of Mild Endometriosis

**DOI:** 10.12669/pjms.39.5.7138

**Published:** 2023

**Authors:** Hui-ling Xue, Wan-jiao Hao, Bing Wang

**Affiliations:** 1 Hui-ling Xue, Department of Obstetrics and Gynecology, Affiliated Hospital of Hebei University, Baoding, Hebei, 071000, P. R. China; 2 Wan-jiao Hao, Department of Obstetrics and Gynecology, Affiliated Hospital of Hebei University, Baoding, Hebei, 071000, P. R. China; 3 Bing Wang, Department of Obstetrics and Gynecology, Affiliated Hospital of Hebei University, Baoding, Hebei, 071000, P. R. China

**Keywords:** Dydrogesterone, Non-steroidal anti-inflammatory, Endometriosis, Treatment

## Abstract

**Objective:**

To evaluate the clinical efficacy of dydrogesterone combined with non-steroidal anti-inflammatory drugs(NSAIDs) in the treatment of patients with mild endometriosis.

**Methods:**

This was a clinical comparative study. Eighty patients with mild endometriosis were recruited at Affiliated Hospital of Hebei University, randomly divided experimental group (n=40) and control group (n=40) from March 2022 to March 2023. Both groups started treatment with dydrogesterone on the 5th day of menstruation. Patients in the control group were treated with dydrogesterone monotherapy, while those in the experimental group were treated with mefenamic acid the basis of the therapy of the control group. The clinical efficacy, differences in the levels of humoral immune indexes, the levels of inflammatory factor and the incidence of adverse drug reactions of the two groups was compared and analyzed.

**Results:**

The efficacy of the experimental group was significantly higher than the control group, with a statistically significant difference(P=0.02). The levels of C3 and C4 in the experimental group after treatment were significantly lower than those in the control group, with a statistically significant difference(P=0.00). After treatment, TNF-a, CRP, IL-6 and other indexes in the experimental group were significantly lower than those in the control group, with statistically significant differences(P=0.00). The incidence of adverse reactions after treatment had no statistically significant difference(P=0.45).

**Conclusion:**

Dydrogesterone combined with non-steroidal anti-inflammatory drugs is a safe and effective treatment for patients with endometriosis. It can improve various obvious curative effects, such as marked relief of pain symptoms, reduction of complement and inflammatory factor levels without a significant increase in adverse reactions.

## INTRODUCTION

Endometriosis is a disease in which the endometrial tissue with growth function appears in other places of the uterus, causing a series of symptoms and signs in patients.^1^ It tends to make inroads on women of childbearing age. Studies suggest that about 5-10%^2^ of women of reproductive age worldwide have suffered from the disease. Endometriosis is prone to occur in the ovary and peritoneum, often accompanied by various degrees of pelvic adhesions. It has a certain impact on patients’ daily life and study, with clinical manifestations such as progressive dysmenorrhea, pelvic pain and dyspareunia.^3^ Clinically, surgery and drugs are usually preferred for the treatment of endometriosis, but postoperative lesions are easy to spread and infiltrate and have a high recurrence rate. Fertility-sparing patients have a disease recurrence rate of approximately 20% within two years after surgery^4^,^5^, and are more likely to damage the urinary system and rectal function of patients. Therefore, patients with mild endometriosis are often treated with conservative drugs.^6^ Gonadotropin-releasing hormone agonist (GnRHa) has been widely used in the treatment of endometriosis.^7^

Despite the exact curative effect, long-term medication may cause a series of symptoms of low estrogen such as vaginal dryness, low-grade fever, and osteoporosis. Dydrogesterone is oral progesterone that is effective in treating endometriosis, such as decreasing endometrial lesions and controlling pain intensity.^8^ Mefenamic acid is a non-steroidal anti-inflammatory drug with a strong analgesic effect and low adverse reactions and risks.^9^ In this study, dydrogesterone combined with mefenamic acid was used to treat patients with mild endometriosis, and certain clinical efficacy was achieved. The details are reported as follows:

## METHODS

This was a clinical comparative study. Eighty patients with mild endometriosis were included at Affiliated Hospital of Hebei University. They were randomly divided experimental group(n=40) and control group(n=40) from March 2022 to March 2023. Patients in the study group were aged 23-57 years, with an average of 37.53±11.42, while those in the control group were aged 22-60 years, with an average of 38.33±12.06. No significant difference was observed in the comparison of general data between the two groups, which were comparable (Table-I).

**Table-I:** Comparative analysis of the general data of the experimental group and the control group (*χ̅*±*S*)n=40.

Index	Experimental group	Control group	t/χ^2^	P
Age (years)	37.53±11.42	38.32±12.06	0.30	0.76
Menstruation cycle	29.43±2.74	29.18±2.30	0.44	0.66
Menstruation	5.37±1.17	5.18±1.20	0.72	0.48
***Lesion sites***			0.09	0.76
Unilateral	34 (85%)	33 (82.5%)		
Bilateral	6 (15%)	7 (17.5%)		
***Staging***			0.22	0.64
Stage I	27 (67.5%)	25 (62.5%)		
Stage II	13 (32.5%)	15 (37.5%)		

P>0.05.

### Ethical Approval:

This study was approved by the medical ethics committee of Ethical Approval: Affiliated Hospital of Hebei University(No.: HDFYLL-KY-2023-026; date: February 28,2023), and written informed consent was obtained from all participants.

### Inclusion criteria:

- Patients who met the diagnostic and treatment criteria for endometriosis^10^;
- Patients with no abnormality in liver and kidney function as well as routine blood and urine routine examinations;
- Patients with a complete medical history and high compliance;
- Patients who have not taken immunosuppressive agents or glucocorticoids in the past three months;
- Patients who themselves or their family members agreed to the study protocol and signed the consent form;
- Patients with complete clinical data;
- Patients with a mass detected by abdominal ultrasound or CT and whose size can be accurately measured;
- Patients with clinical stages I-II.

### Exclusion criteria:

- Patients who have used hormone supplements or hormone drugs in the past three months;
- Patients with severe heart, liver and kidney dysfunction or coagulation dysfunction;
- Patients with uterine fibroids;
- Patients during lactation or pregnancy;
- Patients allergic to the drug in this study;
- Patients with a gastrointestinal ulcer or bleeding;
- Patients with incomplete clinical data;
- Patients with cognitive dysfunction or mental diseases who could not cooperate with the study.

Both groups started treatment with dydrogesterone on the fifth day of menstruationon. Patients in the control group were treated with dydrogesterone monotherapy as follows: 10mg of dydrogesterone twice a day for six months. Those in the study group were combined with oral mefenamic acid on the basis of the control group as follows: 0.25 g of mefenamic acid twice a day, from the first day to the seventh day of menstruation as a course of treatment, usually repeated two to three courses.^11^

Both groups were observed before and after treatment to judge the clinical efficacy^12^: *Markedly effective:* all pelvic masses decreased after treatment, and symptoms such as dysmenorrhea and pelvic pain completely disappeared or improved significantly; *Effective:* all pelvic masses without obvious change after treatment, and symptoms such as dysmenorrhea and pelvic pain improved; *Ineffective:* all pelvic masses did not shrink or even increased after treatment, and symptoms such as dysmenorrhea and pelvic pain did not improve. Total effective rate = (markedly effective + effective)/total number of cases X 100%. Not only the differences in the levels of humoral immune indexes IgG and IgM as well as complements C3 and C4, but also the levels of inflammatory factors between the two groups after treatment and the incidence of adverse drug reactions in the two groups was compared and analyzed.

### Statistical analysis:

All data in this study were statistically analyzed by SPSS 20.0 software, and measurement data were expressed as (*X̅*±*S*). Two independent sample t-test was used for comparison between groups, paired t test was used to analyze data within groups, and χ^2^ test was used for the comparison of rates. P<0.05 indicates a statistically significant difference.

## RESULTS

The comparative analysis of clinical efficacy showed that the efficacy of the experimental group was 90%, which was significantly higher than 70% of the control group, with a statistically significant difference (P=0.02) (Table-II).

**Table-II:** Comparative analysis of clinical efficacy of the two groups (*χ̅*±*S*) n=40.

Group	Markedly effective	Effective	Ineffective	Total effective rate
Experimental group	21	15	4	36 (90%)
Control group	17	11	12	28 (70%)
*χ^2^*				5.00
*P*				0.02

P<0.05.

No statistically significant difference was observed in the levels of IgG and IgM before and after treatment between the two groups (P>0.05); The levels of C3 and C4 in the experimental group after treatment were significantly lower than those in the control group, with a statistically significant difference (P=0.00) (Table-III).

**Table-III:** Comparative analysis of differences in IgG, IgM and complements C3 and C4 between the two groups before and after treatment (*χ̅*±*S*) n=40.

Observation indexes		Experimental group	Control group	t	p
IgG (g/L)	Before treatment	10.53±1.31	10.05±1.27	1.66	0.10
	After treatment	11.44±1.26	11.17±1.18	0.96	0.34
IgM (g/L)	Before treatment	1.53±0.32	1.55±0.30	0.28	0.77
	After treatment	1.90±0.18	1.84±0.22	1.41	0.16
C3 (g/L)	Before treatment	0.98±0.13	0.96±0.11	0.74	0.46
	After treatment*	0.57±0.21	0.78±0.18	4.82	0.00
C4 (g/L)	Before treatment	0.45±0.09	0.47±0.07	1.11	0.27
	After treatment*	0.31±0.05	0.40±0.06	7.28	0.00

*P<0.05.

No statistically significant difference was observed in the comparison of TNF-a, CRP, IL-6 and other indexes between the experimental group and the control group before treatment (P>0.05). After treatment, TNF-a, CRP, IL-6 and other indexes in the experimental group were significantly lower than those in the control group, with statistically significant differences (P=0.00) (Table-IV). The incidence of adverse reactions after treatment in the experimental group was 30%, while that in the control group was 22.5%, with no statistically significant difference (P=0.45) (Table-V).

**Table-IV:** Comparative analysis of changes in inflammatory factors before and after treatment between the two groups (*χ̅*±*S*) n=40.

Indexes		Experimental group	Control group	t	p
TNF-ɑ (ng/L)	Before treatment	23.47±5.72	23.51±6.08	0.74	0.36
	After treatment*	12.01±5.68	15.47±5.36	2.80	0.00
CRP(mg/L)	Before treatment	22.73±7.12	22.85±7.10	0.27	0.76
	After treatment*	11.78±3.07	15.94±4.07	5.16	0.00
IL-6 (ng/L)	Before treatment	12.03±3.16	11.89±2.92	0.33	0.72
	After treatment*	5.28±1.24	7.33±1.25	7.36	0.00

* P<0.05.

**Table-V:** Comparative analysis of the incidence of complications between the two groups (*χ̅*±*S*) n=40.

Group	Fever	Gastrointestinal reactions	Allergy	Insomnia	Fatigue	Hot flashes	Incidence rate
Experimental group	2	4	2	1	2	1	12 (30%)
Control group	3	0	2	0	2	2	9 (22.5%)
*χ^2^*							0.58
*p*							0.45

P>0.05.

## DISCUSSION

It has been confirmed in our study that the efficacy of the experimental group was 90%, which was significantly higher than 70% of the control group, with a statistically significant difference (P=0.02); The levels of C3 and C4 in the experimental group after treatment were significantly lower than those in the control group, with a statistically significant difference (P=0.00). After treatment, TNF-a, CRP, IL-6 and other indexes in the experimental group were significantly lower than those in the control group, with statistically significant differences (P=0.00). The incidence of adverse reactions after treatment in the experimental group was 30%, while that in the control group was 22.5%, with no statistically significant difference (P=0.45).

Endometriosis (EMs) refers to ectopic endometrial tissue that occurs outside the uterine cavity. It is a common benign inflammatory disease in women of childbearing age^13^ characterized by dysmenorrhea, chronic pelvic pain, and infertility, which seriously affects the quality of life of patients.^14^ Presently, endometriosis is still unclear in terms of pathogenesis, and there are mainly several theories about it: excessive estrogen biosynthesis, estrogen-dependent inflammation, and insufficient production and action of vitamin A^15^, among which inflammatory factors are the key factors in the occurrence and development of the disease. In past clinical practice, laparoscopic surgery is a commonly used treatment for endometriosis. Despite being a benign disease, it has the biological behavior of malignant tumors.^16^ Surgery on patients with endometriosis removes only the visible lesions but does not address the underlying cause of the disease. Therefore, such patients can be temporarily relieved of symptoms, but have a high postoperative recurrence rate. The long-term control of endometriosis still relies on drugs, so drug therapy is the first choice for treatment.^17^ Therefore, how to effectively relieve pain and reduce recurrence has become a clinical research hotspot.^18^

EMs is an estrogen-dependent disease that if treated with high-dose progesterone alone, the release of pituitary gonadotropin will be inhibited, resulting in a decrease in estrogen levels; On the other hand, progesterone can also directly act on the endometrium and ectopic endometrium to make endometrium atrophy, resulting in high progesterone amenorrhea and endometrium decidualization.^19^ Studies have shown^20^ that progesterone also has a variety of anti-inflammatory effects in vitro and in vivo, which inhibit the implantation and growth of the endometrium in the retrograde menstrual blood, and inhibit the expression of matrix metalloproteinases and angiogenesis, thus reducing the inflammatory state caused by the metabolic activity of the ectopic endometrium. It was reported that progesterone treatment of EMs^21^ also affects bone metabolism and reduces bone density, but it is far less effective than GnRHa drugs. It has also been shown in a study that dydrogesterone evidently improved the symptoms of pelvic pain caused by EMs.^22^

Hormone therapy for EMs-related pain is only effective in a subset of patients, who often experience recurrence of these symptoms after discontinuation of the drug.^23^ EMs has been shown to potentially induce a local inflammatory response with the recruitment of macrophages, release of cytokines, and production of reactive oxygen species, resulting in a pro-oxidant peritoneal microenvironment. These changes may be systematically reflected to affect the follicular microenvironment.^24^ Cycloxygenase (COX), also known as prostaglandin synthetase, is a key enzyme that catalyzes the conversion of arachidonic acid to prostaglandin. Under the activation of various physical, chemical and biological damage factors, phospholipase A2 hydrolyzes cell membrane phospholipids to generate arachidonic acid, which is catalyzed by COX-2 and oxygenated to generate prostaglandins.^25^ In endometriotic tissues, the expression level of COX-2 is increased.^26^ Studies have shown that non-steroidal anti-inflammatory drugs (NSAIDs) are effective in relieving pain caused by endometriosis^27^, mainly by inhibiting COX-2 and thereby inhibiting prostaglandin synthesis. But at the same time, a variety of adverse reactions may also be caused, such as gastric ulcers, and inhibition of ovulation when taken in the middle of menstruation. In recent years, an increasing number of studies have been carried out on the use of COX-2-specific inhibitors for the treatment of endometriosis pain.

### Limitations:

It includes a small number of cases were included, the follow-up time was short, and some other non-steroidal anti-inflammatory drugs such as ibuprofen were not included in the study for analysis. In response to this, more samples will be included in future studies, the follow-up time will be extended, and the research content of other non-steroidal anti-inflammatory drugs will be increased. It is hoped that with the help of this type of research, more women will be relieved of pain, and their quality of life and fertility will be improved.

## CONCLUSION

Dydrogesterone combined with non-steroidal anti-inflammatory drugs is a safe and effective treatment for patients with endometriosis. It boasts various obvious curative effects, such as marked relief of pain symptoms, and reduction of complement and inflammatory factor levels without a significant increase in adverse reactions.

### Authors’ Contributions:

**HX and WH** carried out the studies, participated in collecting data, and drafted the manuscript, and are responsible and accountable for the accuracy or integrity of the work. **WH and BW** performed the statistical analysis and participated in its design. All authors read and approved the final manuscript.
